# Conflicting phylogenetic signals in the SlX1/Y1 gene in Silene

**DOI:** 10.1186/1471-2148-8-299

**Published:** 2008-10-30

**Authors:** Anja Rautenberg, Dmitry Filatov, Bodil Svennblad, Nahid Heidari, Bengt Oxelman

**Affiliations:** 1 Department of Systematic Biology, EBC, Uppsala University, Sweden; 2 Department of Plant Sciences, University of Oxford, UK; 3 Mathematical Statistics, Department of Mathematics, Uppsala University, Sweden; 4 Department of Plant and Environmental Sciences, University of Gothenburg, Sweden

## Abstract

**Background:**

Increasing evidence from DNA sequence data has revealed that phylogenies based on different genes may drastically differ from each other. This may be due to either inter- or intralineage processes, or to methodological or stochastic errors. Here we investigate a spectacular case where two parts of the same gene (*SlX1*/*Y1*) show conflicting phylogenies within *Silene (Caryophyllaceae)*. *SlX1 *and *SlY1 *are sex-linked genes on the sex chromosomes of dioecious members of *Silene *sect. *Elisanthe*.

**Results:**

We sequenced the homologues of the *SlX1*/*Y1 *genes in several *Sileneae *species. We demonstrate that different parts of the *SlX1/Y1 *region give different phylogenetic signals. The major discrepancy is that *Silene vulgaris *and *S*. sect. *Conoimorpha *(*S. conica *and relatives) exchange positions. To determine whether gene duplication followed by recombination (an intralineage process) may explain the phylogenetic conflict in the *Silene SlX1/Y1 *gene, we use a novel probabilistic, multiple primer-pair PCR approach. We did not find any evidence supporting gene duplication/loss as explanation to the phylogenetic conflict.

**Conclusion:**

The phylogenetic conflict in the *Silene SlX1/Y1 *gene cannot be explained by paralogy or artefacts, such as *in vitro *recombination during PCR. The support for the conflict is strong enough to exclude methodological or stochastic errors as likely sources. Instead, the phylogenetic incongruence may have been caused by recombination of two divergent alleles following ancient interspecific hybridization or incomplete lineage sorting. These events probably took place several million years ago. This example clearly demonstrates that different parts of the genome may have different evolutionary histories and stresses the importance of using multiple genes in reconstruction of taxonomic relationships.

## Background

One of the challenges of evolutionary biology is phylogeny reconstruction. Modern techniques have facilitated the use of DNA sequences as the primary source of phylogenetic data. Usually, only small fractions of the genome are analyzed. In systematic research, such regions are often assumed to reflect the organismal lineage ("species") phylogeny. In principle, however, the resulting phylogenies do not reflect the history of the species, but rather the history of the individual DNA regions themselves (e.g. [1]). These regions may have different evolutionary histories, which can lead to presence of several conflicting gene phylogenies. Taken together, these phylogenies can give clues to the organismal phylogeny (e.g. [2]). Incongruent gene phylogenies may have different causes, e.g. introgression (e.g. [3,4]), homo- or polyploid hybridization (e.g. [5,6]), mistaken orthology due to gene duplications and losses [7], or incomplete lineage sorting of alleles [8]. In fact, coalescence theory predicts that in some cases, the most probable gene tree will not even reflect the species tree [9,10], and this has also been shown in simulation studies using coalescent models on concatenated data [11].

In order to understand how gene phylogenies relate to organismal phylogenies, it is important to obtain sequence data from different parts of the genomes [12,13]. Plant molecular phylogenetics has heavily utilized chloroplast and nuclear ribosomal DNA (nrDNA) data, whereas other parts of the nuclear genome, as well as the mitochondrial genome, are much less utilized. To differentiate between different causes of gene tree discordances, the use of multiple, potentially unlinked low-copy gene regions is desirable (e.g. [12-15]).

If sampling of a multi-copy gene family is poor, paralogues may be misidentified as orthologues [7]. Paralogy problems may, however, exist even if entire genome sequences are at hand, if paralogues have gone extinct haphazardly in different lineages [16]. To complicate matters even further, members of a multi-copy gene family, and also alleles of the same gene, may recombine and make phylogenetic analysis difficult [7]. Paradoxically, this property has facilitated the use of nrDNA sequences, since cistrons from this gene family often are present in very large tandemly repeated numbers. These copies are usually very similar due to the process of concerted evolution, operating by e.g. unequal crossing over and gene conversion (e.g. [17,18]).

**Figure 1:**
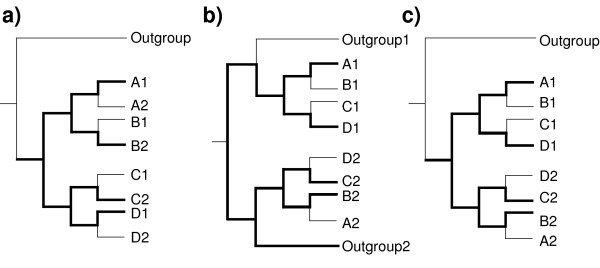
**Possible outcomes of incomplete paralogue sampling**. Possible outcomes of incomplete paralogue sampling (bold lines) in relation to different relative ages of gene duplications. a) Recent gene duplications. The copies (inparalogues) are monophyletic within terminal taxa (e.g. organisms). The relationships among these remain unchanged, regardless of which copies are included in the study. b) An early gene duplication has created two ancient outparalogues. If different paralogues are sequenced for different taxa, the ingroup will not become monophyletic. c) A gene duplication has created two recent outparalogues. Organismal phylogenetic inference will give erroneous results, if some of the paralogues remain undetected. A through D represent ingroup taxa, numbers indicate sequence copies.

To distinguish intralineage processes such as gene duplications from interlineage processes (e.g. hybridization) causing tree discordances, we are mainly interested in gene copies at three different levels. We follow the terminology of [19] and use the term "inparalogues" for genes resulting from duplications within terminal taxa/lineages (Figure 1a). Duplications that occur along the internal branches of the ingroup species tree will be referred to as "recent outparalogues" (Figure 1c, cf. [19]). Genes that have duplicated before the origin of the group of interest will be referred to as "ancient outparalogues" (Figure 1b). "Sequence copies" or "sequence variants" means any kind of alleles, paralogues or orthologues that are similar enough to be aligned with each other.

In organisms where complete genome sequences are not available, paralogy determination has to be considered by heuristic methods. A classical method to find paralogues is to use Southern blot hybridization [20]. A drawback of this method is that it only gives a number of bands able to hybridize with the probe under specific conditions. It does not reveal the actual sequences, which makes it impossible to make any detailed assertions about the homology status of the different bands detected.

Another way to examine paralogues is to use a phylogenetic approach to sort out the different copies of the gene. This has been applied to genomes with complete sequence data (e.g. [21]). To deal with genomes with incomplete sequence information, Small & al. [15] suggested a combination of PCR-mediated sequencing, Southern blots, and expression studies. They argue that the sequence-based approach is the weakest and that especially Southern blots can be used to strengthen orthology assessment. However, they did not consider using multiple PCR primer pairs to amplify several overlapping DNA fragments instead of amplifying one single fragment.

While the combination of the approaches certainly is powerful, we think that there is room for improvement of the PCR-mediated approach. In principle, multiple PCR primers could be used with essentially the same coverage as constructed hybridization probes, but with the difference of being more sensitive and, most importantly, giving access to the actual sequences and thus to sophisticated phylogenetic analyses. A major advantage here is that gene trees derived from sequence data provide a possibility to assess the detailed paralogy status (in- or outparalogues of different relative ages) of multiple sequence copies. A weakness of the PCR method, however, is that some sequence copies may be preferred in the amplification (PCR bias, e.g. [22]). Using multiple primer pairs along the sequence might alleviate this problem to some extent, but if these primers were designed from a single sequence, they will on average be biased towards sequences that are similar to the template sequence. To circumvent this problem, one can design primers placed in more conserved parts of the sequence. Obviously, this requires some knowledge of the sequence diversity present. Although difficult to quantify exactly, PCR bias will decrease and the possibility to find paralogues will increase with the number of independent PCR primer pairs used.

To assess the efficiency of a multiple PCR-primer method, we use a probabilistic approach. Under the assumption that different primer pairs are independent and that they do not, on average, preferentially prefer some copies to others, we can calculate the probability that all sequence copies have been detected with these primer pairs.

In order to use nuclear genes in phylogenetic analyses when entire genome sequence information is not at hand, it is often necessary to develop protocols that are specific for the plant group in question [14]. Popp & Oxelman [13] developed a protocol to use RNA polymerase genes in phylogenies, which has been successfully used in subsequent *Sileneae (Caryophyllaceae) *studies to reveal a number of conflicting gene trees ([5,23], unpublished data). Here, we test the utility of another low-copy nuclear gene, *SlX1/SlY1*. *SlY1 *is a sex-linked gene described from the Y chromosome of *Silene latifolia *Poir., a dioecious member of *Silene *sect. *Elisanthe *(Fenzl ex Endl.) Ledeb. [24]. *SlX1 *is a closely related gene, located on the X chromosome [24,25]. There are also homologues in the other dioecious species in *Elisanthe *[26,27] and on the autosomes in non-dioecious taxa [26-29]. The region is hereafter called "XY1". An initial Southern blot study suggested that there may be several paralogues of XY1 [24], but in later PCR-based studies, only a single XY1 copy has been found [28,29].

Preliminary phylogenetic analyses of XY1 sequences indicated that different parts of the gene give rise to conflicting *Silene *phylogenies. These conflicts mainly involved the relationships between a few distinct lineages: *Silene *sections *Elisanthe *and *Conoimorpha *and some of their relatives in *Silene *subgenus *Behenantha *(Otth) Endl. (= subgenus *Behen *sensu e.g. [13]). The dioecious species in sect. *Elisanthe (S. latifolia, S. dioica *(L.) Clairv., *S. diclinis *(Lag.) Lainz, *S. heuffelii *Soó, *S. marizii *Samp.) are characterized by sexual dimorphism and sex chromosomes. Previous studies indicate a close relationship between the dioecious species in *Silene *sect. *Elisanthe *and the members of *Silene *sect. *Conoimorpha *Otth [30-34], The members of the *Conoimorpha *group all have calyces with several (up to 60) prominent parallel nerves. *Silene vulgaris *(Moench) Garcke represents a group of species having strongly inflated calyces with reticulate nerves. They appear closely related to the annual *S. behen *L. (with which it share some morphological characteristics) and also to *S. pendula *L. [30,33].

The aim of this study is to investigate the historical explanation to a case where different parts of a gene (XY1) give rise to conflicting phylogenies within *Silene*. To investigate whether gene duplication/loss may be a plausible explanation we present a novel probabilistic PCR approach to determine the number of sequence variants present in an organism.

## Results

### Number of sequence variants

In *S. conica *L., *S. conoidea *L. and *S. pendula *only one XY1 sequence variant was found (see Table 1 for voucher information). In *S. acaulis *L. two variants were found and in *S. nutans *L. and *S. vulgaris *three variants were found (Table 2). Using our novel approach to calculate the posterior probability that the actual number of sequence variants is the observed number (achieved from different independent, partially overlapping PCR products, and using a discrete uniform distribution as prior), we found that for all species except *S. nutans*, the posterior probability is > 0.99 that there are no more undetected variants (Table 2. See Methods for explanation and formula). The divergence between the variants within an individual was always less than the divergence between sequences from different species (Table 2). The entire alignment of the XY1 region contained 6416 bp and 361 indel characters. The reduced 9-taxon alignment, where parts with large amounts of missing data (due to long indels or unsequenced regions) in many sequences were excluded, contained 4045 bp.

**Table 1:** Vouchers used for sequencing of SlX1/SlY1/SlXY1 genes

Taxon	Group	DNA type	Voucher (or reference)	Accession
***S. acaulis *(L.) Jacq**.	**S**	**XY**	**Oxelman 2419 (UPS)**	[EMBL:FM204668]^A^, [EMBL:FM204669]^B^
***S. conica *L**.	**B, C**	**XY**	**Erixon 70 (UPS)**	[EMBL:FM204663]
*S. conica*	B, C	XY, mRNA	Filatov & Charlesworth 2002[28]	[EMBL:AY082378]
***S. conoidea *L**.	**B, C**	**XY**	**Rautenberg 290 (UPS)**	[EMBL:FM204664]
*S. dioica *(L.) Clairv.	B, dE	X	Filatov & Charlesworth 2002[28]	[EMBL:AY084044]
*S. dioica*	B, dE	Y	Filatov & Charlesworth 2002[28]	[EMBL:AY084045]
*L. flos-jovis *(L.) Desr.	L	XY	Filatov & Charlesworth 2002[28]	[EMBL:AY084042]
*S. latifolia *Poir.	B, dE	X, ♂plant	Delichère & & al. 1999[24]	[EMBL:AJ310656]
*S. latifolia*	B, dE	X	Filatov & Charlesworth 2002[28]	[EMBL:AY084036]
*S. latifolia*	B, dE	Y	Filatov & Charlesworth 2002[28]	[EMBL:AY084037]
*S. latifolia*	B, dE	Y, ♂ plant	Delichère & al. 1999[24]	[EMBL:AJ310655]
*S. noctiflora *L.	B, E	XY, mRNA	Nicolas & al. 2004[27]	[EMBL:AJ631222]
***S. nutans *L**.	**S**	**XY**	**Larsen, Larsen & Jeppesen 196 (S)**	[EMBL:FM204670]^A^, [EMBL:FM204671]^A^, [EMBL:FM204672]^B^, [EMBL:FM204673]^B^, [EMBL:FM204674]^C^, [EMBL:FM204675]^C^
***S. pendula *L**.	**B, Be**	**XY**	**Rautenberg 289 (UPS)**	[EMBL:FM204662]
*S. vulgaris *(Moench) Garcke	B, Be	XY	Filatov & Charlesworth 2002[28]	[EMBL:AY084040]
***Silene vulgaris ssp. angustifolia *(Miller) Hayek**	**B, Be**	**XY**	**Thulin 5717 (UPS)**	[EMBL:FM204665]^A^, [EMBL:FM204666]^B^, [EMBL:FM204667]^C^

Groups are according to Oxelman & Lidén [31], and Oxelman & al. [13,33], where B = *Silene *subgenus *Behenantha *(Otth) Endl., Be = *Silene *sect. *Behenantha *Otth, C = *Silene *sect. *Conoimorpha *Otth, E = *Silene *sect. *Elisanthe *(Fenzl ex Endl.) Ledeb., dE = dioecious *Silene *sect. *Elisanthe*, L = *Lychnis *L., S = *Silene *subgenus *Silene*. Note that Be, C, and E are nested within B. "DNA type" indicates type of sequence (XY = *SlXY1*, from autosomes, X = *SlX1 *from X chromosome, Y = *SlY1*, from Y chromosome, ♀ = female plant, ♂ = male plant, mRNA = only exons). Specimens in bold were sequenced in the present study. For these accessions voucher information (collector, number and herbarium abbreviations, according to Holmgren & al. [61]) is given. More voucher details can be found in *Sileneae *database [62]. For other accessions references to original publications are given. Accession numbers in GenBank/EMBL. Superscripts A, B, C correspond to sequence names in the phylogenies.

**Table 2:** Number and length of XY1 sequences

Taxon	*S. acaulis*	*S. conica*	*S. conoidea*	*S. nutans*	*S. pendula*	*S. vulgaris ssp. angustifolia*
Number of copies (*x*_*obs*_)	2	1	1	3	1	3
Length (bp)	4517/4534	6280	6036	3004/3009/2979 + 1463/1459/1459	6738	2150 + 1890/3894/4618
Covered area (exon numbers)	2–15	1–15	1 (intron)-15	3 (intron)-6 (intron) + 10–14 (intron)	1–14	1 (intron)-14
Included PCR fragments *(n)*	14	8	9	3	9	17
P	0.9965	0.9959	0.9980	< 0.95	0.9980	0.9924
Distance between copies (± S E)	0.001 ± 0.001			0.004 ± 0.001		0.024 ± 0.002
Mean distances to other taxa ± SE	0.045 ± 0.004–0.125 ± 0.008			0.045 ± 0.004–0.128 ± 0.007		0.064 ± 0.004–0.123± 0.009 (distance to the other vulgaris sequence = 0.032 ± 0.002)

Number and length of XY1 sequence copies found. Indicated are also which parts of the region that are covered in which taxa. The *"n" *row indicates number of PCR fragments included in the probabilistic calculations of sequence numbers. The "p" row indicates the posterior probabilities that we did find all sequence copies (see Methods for formula). The divergence estimates are means from pairwise Tamura-Nei distances ± standard error (SE) from sequences within an individual, and mean distances to sequences from other individuals.

### Conflicting phylogenetic signals in different parts of the alignment

**Figure 2:**
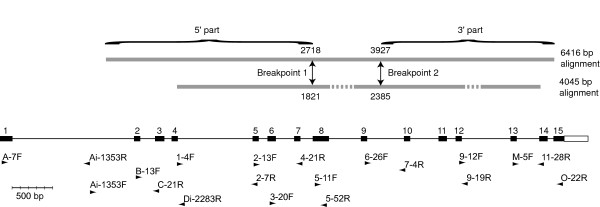
**Included parts of XY1**. Thick grey lines indicate parts of the XY1 region included in the long (upper line) and reduced (lower line) alignments. Dotted lines indicate parts that were excluded. Recombination breakpoints suggested from the GARD analysis are indicated with arrows. The black boxes indicate exons, thin lines introns. The PCR primer positions are indicated below by arrows (sequence-specific primers not included). The intron/exon figure is redrawn from Atanassov & al. [29], and the lengths are based on *S. latifolia *Y1 sequences.

**Figure 3:**
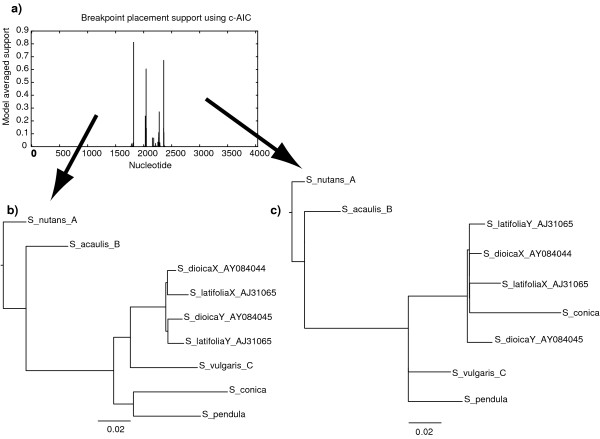
**Results from the GARD analysis**. a) Support for the suggested positions of breakpoints in GARD analysis using the HKY85 nucleotide substitution model and beta-gamma rate distribution with 5 rate classes on the reduced XY1 matrix (4045 bp), Neighbor-Joining trees for the 5' (b) and 3' non-recombinant partitions (c).

The GARD recombination detection screening suggested several recombination breakpoints in the reduced 4045-bp alignment, resulting in two larger non-recombinant partitions in the alignment. The central part of the alignment was divided into a few short partitions by additional recombination breakpoints. Different analysis settings resulted in variation in the number and placement of breakpoints. The positions of the outermost breakpoints, however, differed only slightly: directly before exon 8 and in the intron between exons 9 and 10 (Figure 2, Figure 3, Table 3). The main differences between the partitions (hereafter referred to as the 5' and 3' parts, respectively) are that *S. vulgaris *and *S. conica *change places in the position closest to the dioecious species *S. latifolia *and *S. dioica *(Figure 3).

**Table 3:** Breakpoint locations

	5' part	3' part	Entire alignment
Positions in reduced alignment	1–1821	2385–4045	1–4045
Corresponding positions in full alignment	1–2718	3927–6416	1–6416
Number of base/indel characters	2718/148	2490/150	6416/361
Substitution model	GTR + Γ	GTR + Γ	GTR + Γ

Locations of breakpoints between non-recombinant parts of the reduced 4045-bp alignment suggested by GARD, and corresponding positions in the longer alignment. Substitution models were suggested by MrModeltest AIC.

#### Bayesian phylogenies

**Figure 4:**
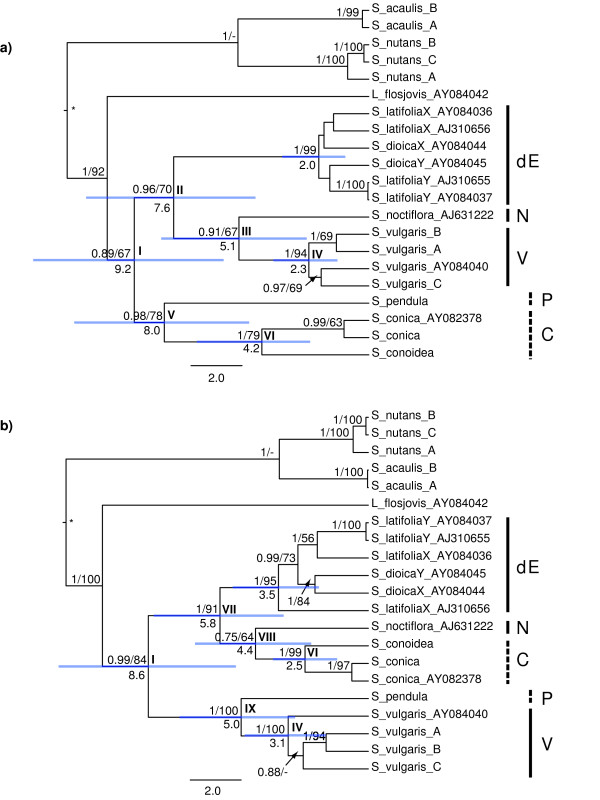
**Phylogenies based on different parts of the XY1 region**. Bayesian consensus chronograms for the 5' (a) and 3' parts (b) of the 6416 bp XY1 alignment. The partitions in a) and b) represent non-recombinant parts that were suggested by GARD from a reduced 9-sequence 4045 bp alignment of the XY1 gene. Values below branches are median ages in million years. Numbers above branches are posterior probabilities (PP)/maximum parsimony bootstrap (MPB) support values for the same alignment but with additional indel characters (values of PP < 0.70 and MPB < 60% are not shown). Hyphens (-) indicate nodes with high posterior probabilities that were not present in the MPB trees. Note that the MBP trees are unrooted, and there is thus only a single internal branch connecting the clades where the BEAST root (*) appears. Horizontal bars represent 95% HPD age intervals. Roman numbers are used to label nodes. Note how the positions of the groups sect. *Conoimorpha *(C) and *S. vulgaris *(V) change in the different partitions.

Applying Bayesian phylogenetic methods to the partitions suggested by GARD on the full 6416 bp alignment with more taxa (Figure 4) resulted in trees compatible to the Neighbor-Joining trees from GARD. In the three cases (*S. acaulis*, *S. nutans*, *S. vulgaris*) where there were more than one sequence variant per individual, these were always monophyletic within the species (inparalogues). *Silene conica *and *S. conoidea*, representing sect. *Conoimorpha*, grouped together with strong support (Figure 4). Also *S. dioica *and *S. latifolia *constituted a well-supported group (dioecious *Elisanthe) *in both partitions (Figure 4). *Silene noctiflora *L., the type species of sect.*Elisanthe *[35], did not form a monophyletic group with the dioecious species (Figure 4).

In the 5' part of the alignment *S. vulgaris *grouped together with *S. noctiflora *as a sister-group to the dioecious species in sect. *Elisanthe*. This whole clade (II) was sister to a clade with the *Conoimorpha *group and *S. pendula *(Figure 4a). In the 3' part of the alignment, sect. *Conoimorpha *grouped together with *S. noctiflora *as the closest relatives (VI) to the dioecious *Elisanthe. Silene vulgaris *grouped with *S. pendula *(Figure 4b).

The ages of the nodes including *S. vulgaris *and its closest relatives were 5.1 million years in the 5' part (split with *S. noctiflora)*, and 5.0 million years in the 3' part for the split with *S. pendula *(Figure 4). The corresponding ages for sect. *Conoimorpha *were 8.0 million years in the 5' part (split with *S. pendula) *and 4.4 million years in the 3' part (split with *S. noctiflora) *(Figure 4). However, the 95% HPD intervals for these nodes overlap considerably.

#### Parsimony phylogenies

The parsimony trees for the two partitions were congruent with the corresponding trees obtained by Bayesian inference and by the GARD analysis, both using the reduced alignment (data not shown) and the full 6416-bp data set with additional gap characters (MPB values in Figure 4).

## Discussion

*Silene vulgaris *and the monophyletic group *Conoimorpha *exchange positions in our phylogenetic trees somewhere between the first 2.7 kbp and the last 2.5 kbp of the 6416 bp XY1 alignment (Figures 3, 4). The last 2.5 kbp gives a phylogeny in agreement with those obtained by the nuclear ribosomal DNA region *ITS *[30-32] and the low-copy nuclear genes *RPA2*, *RPB2 *and the *RPD2a *gene (Rautenberg & Oxelman, unpublished data). Also chloroplast DNA rps16 [33] and a large chloroplast DNA data set [34] show agreeing phylogenies. In contrast, the first 2.7 kbp of the alignment gives an unexpected phylogeny, where *S. vulgaris *is placed closely related to the dioecious *Elisanthe *species. This deviating phylogenetic pattern was also recently found in the *RPD2b *gene (Rautenberg & Oxelman, unpublished data). The results from the XY1 gene agree with previous studies that have shown that *S. noctiflora *does not belong with the dioecious species in *Silene *sect. *Elisanthe *[30-33,36].

Conflicts between separate phylogenies based on different genes can be observed on several genomic levels: when comparing phylogenies based on different genomes (e.g. organellar vs. nuclear), different genes or different copies of a gene (either alleles or paralogues), or even different parts of one gene. These conflicting phylogenies can either reflect complex phylogenetic patterns (e.g. [7,23]), or simply highlight errors and problems in lab procedures and/or phylogenetic methods. We will here discuss possible causes of the observed phylogenetic conflict.

### Lab errors and artefacts

• *Contamination or mix-up of extractions, PCR samples or sequence reactions: *The sequences are verified by multiple accessions of the species, and multiple PCR and sequence reactions. The data set also includes sequences made in different laboratories from different source plants. Thus, this possibility can be safely rejected.

• *Recombination between paralogues or alleles during PCR*: Paralogous gene copies or alleles can recombine during PCR (e.g. [37-39]). This process could create a pattern like the observed one, if our sequencing reactions would have been based on single PCR products of the entire gene. However, our PCR products include several overlapping fragments, 700–2500 bp long.

• *Inconsistent alignment procedures *in separate parts of the alignment could also influence the accurateness of the resulting phylogeny. This explanation is also unlikely, because the included alignment appears unproblematic. Two 700 bp parts of the alignment with much indel variation were excluded in the reduced alignment used in the GARD analysis.

• *Inappropriate phylogenetic methods or sampling errors: *Choosing the wrong phylogenetic method is a potential cause of inconsistent phylogenies. In this study, we used distance methods, Bayesian methods and parsimony methods for phylogeny reconstruction. The same general pattern was found with all methods. The different methods applied here have very different theories and potential problems [40], and in addition, the bootstrap support values and the posterior probabilities are high. Therefore, we argue that the observed pattern is not likely to have been caused by inappropriate methodologies. The high support values also make stochastic errors (i.e. unfortunate sampling of substitution patterns along the sequence alignment) an unlikely explanation.

### Biological explanations

• *Existence of paralogues*: If a gene duplicates within a lineage, each of the resulting paralogues will have its own evolutionary fate. Difficulties in orthology determination can seriously distort phylogenies and conclusions drawn from them. Two processes are potentially serious when inferring organismal phylogenies from multi-copy sequences. First, *in vivo *recombination between the gene copies may give rise to mosaic sequences, which can give inconsistent phylogenies, where different parts of the alignment will reflect conflicting topologies. Second, there is a risk that orthology is mistaken in the phylogenetic analysis if some of the existing outparalogues are not detected, or if some of the paralogues are lost in some lineages. The phylogeny will then be influenced by which copy is lost (or not detected) in which lineage [16]. On the other hand, inparalogues (gene copies that are monophyletic within an individual) will not cause such problems (Figure 1a). Ancient gene duplications (earlier than the divergence of the studied organismal group) followed by haphazard losses will lead to a pattern with some parts of the ingroup grouping with the outgroup (i.e. the ingroup will appear non-monophyletic, Figure 1b). This effect will, under the assumption that the in- and outgroups are correctly circumscribed, indicate that there is a paralogy problem. Thus, only recent outparalogues, i.e. genes that have duplicated after the divergence of the ingroup, but before the origin of the terminal taxa (leaves), are of serious concern for orthology/paralogy interpretation in our case (Figure 1c).

Recombination between two or more paralogues could certainly create a pattern like the one we observe. Results from Southern blot hybridization experiments made by Delichère & al. [24] suggest that there may be one or more copies of *SlX1/SlY1 *on the chromosomes of *S. latifolia*. It is unknown whether these extra copies are inparalogues, recent outparalogues, or if their origins are more ancient than the origin of *Silene *(ancient outparalogues). In *S. conica*, the gene orthologous to *SlX1/SlY1 *seem to be single copy according to our results and those of Atanassov & al. [29]. RT-PCR experiments by Nicolas & al. [27] also revealed only one sequence in all dioecious species analyzed. The within-species sequence variation we observed in *S. vulgaris, S. acaulis *and *S. nutans *is possibly caused by allele variation and/or recent gene duplication, resulting in inparalogues. A natural explanation to the multiple bands detected in some of the Southern blots experiments [24] might therefore be ancient outparalogues that diverged before the diversification of *Silene*, or other genes with a similar sequence. However, an alternative explanation might be that they represent silent pseudogenes that have diverged so much that our PCR experiments have failed to target them. Although not an impossible explanation, we consider this to be unlikely, given the large number of different PCR primer pairs used by us on taxa representing various major lineages in *Silene *and given the high posterior probabilities from the statistical calculations. Note however that relaxation of selective constraints in pseudogenes may result in elevated substitution and indel rates, resulting in violation of the assumption of non-biased targeting of the primers constructed from an alignment of apparently functional gene sequences.

• *Incomplete lineage sorting*: In recently diverged lineages, the alleles from the ancestral gene pool might not yet have become sorted into the new lineages. One way to reject incomplete lineage sorting as a possible cause of incongruence could be to compare the divergence times of the conflicting nodes. Assuming that one of the trees does reflect the organismal tree, lineage sorting can be rejected if the divergence time of the organismal tree node is older than in the deviating tree. If we consider the tree from the 3' part of XY1 to be the most likely organism tree (as is supported by other data, see above), we cannot reject incomplete lineage sorting because the ages are very similar (node IX versus node III, Figure 4) or considerably younger (node VIII versus node V, Figure 4). However, the 95% HPD intervals for the relevant nodes overlap largely (Figure 4), and we lack a robust hypothesis about dating of splits in the species tree. It is therefore not possible either to reject or corroborate incomplete lineage sorting as the cause of the observed incongruence.

• *Horizontal gene transfer*: Recently, horizontal (or lateral) gene transfer in plants has been reported (reviewed in [41]). Most of these cases are mitochondrial genes that seem to be transferred between isolated lineages, but two examples of horizontal transfer of nuclear genes have also been suggested [42,43]. Although we cannot rule out horizontal gene transfer completely, we do not have a reasonable explanation on the mechanisms and series of events that could create the observed pattern.

• *Hybridization *also creates patterns where different parts of the hybrid's genome reflect relationships with the different parental taxa. If the hybridization is a success, genes or alleles with separate evolutionary histories will become introduced into the offspring. Through repeated backcrossing with one of the parental lineages, only a minority of the other lineage's genes will prevail ("introgression") and the resulting pattern will mimic horizontal gene transfer.

For a hybridization event to be a favourable explanation when groups exchange positions between two trees, the age of the split disagreeing with the species tree should be younger than in the species tree [23]. If we assume that the 3' part of the XY1 alignment reflects the organism tree (as is corroborated by other data), the hybridization explanation is not supported if the ages of nodes VIII and V are taken at face value (Figure 4b). The 95% HPD intervals for the nodes are broad, however, so hybridization cannot be rejected. Nodes IX and III have very similar median ages, making the discrimination between hybridization and intralineage processes even more obscure. Denser taxon sampling could possibly narrow the HPD intervals.

There are no morphological characters suggesting that hybridization has taken place. On the other hand, this putative event probably lies several million years back, and given the rampant morphological homoplasy in *Silene *in general, this is perhaps not surprising.

An enigmatic feature of the phylogenetic results is that the lineages of *S. vulgaris *and the *Conoimorpha *group appear to mutually switch positions in the trees. There is no reason to expect that recombination should take place at the same sequence location in different lineages, either under a paralogy or under a hybridization hypothesis. However, the taxonomic sampling is sparse, and further sampling might reveal that the phylogenetic positions are not mutually exchanged. The fact that GARD actually supports several recombination events may indicate sequential events, rather than a reciprocal switch.

*Lychnis flos-jovis *appears within *Silene *in our trees (Figure 4). The phylogenetic status of *Lychnis *in relation to *Silene *is not strongly supported [32], but recent studies (e.g. [13]) have rather corroborated the sister-group relationship between the two. However, other data (e.g. [23,34]) also indicate complicated patterns that may involve reticulations. Here, we follow Oxelman & al's [32] generic classification of the tribe *Sileneae*, but the purpose of this paper is not to draw any taxonomic conclusions.

A difficulty when working with organisms where the entire genomic sequences are not known is to estimate confidence in whether the number of sequence variants (alleles, paralogues) detected does reflect all variation within the organism. Joly & al. [44] used a binomial distribution to calculate the number of clones from a PCR product that had to be sequenced to achieve a certain probability of sampling all alleles in a tetraploid individual (given that the primer pair picks all variants). Since the binomial distribution assumes the events of finding an allele to be independent we argue that a Bayesian approach is more appropriate. The methods appear to give similar results, however. Our approach has the advantage that the total number of sequence copies does not need to be known. Also, replicating the number of independent PCR primer pairs decreases the risk of PCR bias.

## Conclusion

There is a phylogenetic conflict in different parts of the *Silene SlX1/Y1 *gene that cannot be explained by gene duplications/losses or artefacts, such as in vitro recombination during PCR. This phylogenetic incongruence may have been caused by recombination of two divergent alleles following horizontal gene transfer, interspecific hybridization or incomplete lineage sorting. Given our results of the relative dating, we can reject neither of these hypotheses. However, the fact that we recently discovered a phylogenetic pattern similar to that from the first part of the XY1 alignment also in the *RPD2b *gene (unpublished data) can be interpreted as support for the hybridization/introgression hypothesis.

Our novel probabilistic PCR approach, in combination with phylogenetic methods, provides a useful way to discriminate between different paralogue types and to determine the number of outparalogues in a genome, when the entire genomic sequence is not known.

This example clearly demonstrates that different parts of the genome may tell us different stories and stresses the importance of using multiple genes in reconstruction of taxonomic relationships.

## Methods

### Taxa

Six specimens representing various phylogenetic lineages in *Sileneae *(Table 1) were screened for XY1 sequences using PCR outlined below. In addition, GenBank sequences, including several representatives of *Silene *sect. *Elisanthe *were used (Table 1). Taxa were chosen to mostly include representatives from *Silene *subgenus *Behenantha*. *Lychnis flos-jovis *(L.) Desr. and representatives from *Silene *subgenus *Silene *were used as outgroups [13,31,33]. All included taxa are diploid [45,46]. Genus names follow the generic classification of *Sileneae *by Oxelman & al. [32].

### DNA isolation

Isolation of total genomic DNA was performed from herbarium specimens or fresh material using a modified Carlson/Yoon method [31]. Most DNA isolations were purified by the GFX Purification Kit (Amersham Biosciences) and dissolved in EB buffer (10 mM Tris-Cl, pH 8.5, QiaGen). Some were purified by the Ultra Silica Bead Kit (ABgene).

### Primers and PCR

Primers for the XY1 region were designed to amplify several partially overlapping fragments of XY1 (Figure 2). A preliminary alignment with several *Silene *taxa (GenBank accessions in Table 1 and 10 unpublished sequences with similar sequence diversity as the sequences used in the analyses) was used to get the initial primer sequences. The primers were aimed to work on all of the XY1 variants. Most primers were positioned in exons (Figure 2, Table 4). Details on PCR conditions can be obtained from the first author on request.

**Table 4:** XY1 primers

primer name	primer sequence
A-7F	GGAGGCAAGAAAGCATTGAG
Ai-1353F	GATCACATTTAGGCCAGT
B-13F	CGCCAACGTCTTTATCTCTCA
C-21R	TGGGTTTCACGACTTCAACA
1-4F	AACGATAATACATCCCGGTGAG
Di-2283R	CACAATAGAGAAGCCCAAAGTT
2-13F	GTTGCAACTCATACTGACAGTCC
2-7R	GGAGCTCCCTAATCCTGTTT
3-20F	TCTCGTCCAGATTTGGTGTG
4-21R	AGCGGTTCAGAAGAGCACAT
5-11F	ATAAGTCAGTTGTTTTGTGGAGCATC
5-52R	ATGCCTCGAGGTCCAATAGA
6-26F	AAGAGCTGGATTGACGCCAGTGAC
7-4R	TGAAGATCAGCATTGTGAGCTTTCTC
9-12F	TGCTGAAGATGGCTTGCTAA
9-19R	AAGCCATCTTCAGCAGCACT
M-5F	GGAAACAGAGAGCGGAGGTA
11-28R	CAGCAGAGCTTGAACAGTCATCT
O-22R	CAGCTCAGCCAAAACTTCCT

Sequences for XY1 primers used for PCR (many also for sequencing, sequence specific primers used for sequencing are available on request from the first author). Specific primers are not included.

### Specific primers

When the sequences were polymorphic due to indel polymorphisms, specific primers were designed, either directly from the sequenced PCR products, or from cloned sequences. These new primers were used in later PCR (in a few cases) and for direct sequencing of the polymorphic PCR products.

### Sequencing

Purified PCR products were sent to Macrogen Inc. in Seoul, South Korea for sequencing (using the BigDyeTM terminator kit and run on ABI 3730XL). Some sequence reactions were run on an ABI 3700 sequencer at Rudbeck lab, Uppsala University, Sweden. Sequencing reactions were in this case carried out using the BigDye 3.1 kit.

### Cloning

In some taxa, cloning of PCR products was performed to overcome problems with indel polymorphisms. The PCR fragments were obtained by Taq (ABgene), and were cloned using TOPO TA cloning Kit for Sequencing (Invitrogen), with half the recommended reaction volumes. From each cloning reaction, 7–13 colonies were picked for PCR using the universal primers M13F and M13R. Purified PCR products were sequenced by Macrogen Inc. using the universal primers T3 and T7promoter available at Macrogen Inc. and otherwise as above. Mostly, the cloned sequences were only used as a base for subsequent primer design, but in some cases the actual sequences were also used in the alignment. Single base polymorphisms that occurred only in single clones were considered as PCR artefacts and discarded from subsequent analyses.

### Assembly and alignment

The Staden package version 1.6.0 for Mac OS X [47] with phred version 0.020425.c and phrap version 0.990319 [48] was used to assemble readings into contigs. In some cases manual editing of the contigs was made. Base polymorphisms were coded using the NC-IUPAC ambiguity codes.

The resulting contig sequences were aligned manually using QuickAlign [49], using the criteria of Popp & Oxelman [13]. The sequences were trimmed to reduce the number of taxa with long stretches of missing data in the beginning and end. Parts of the introns between exons 8/9 and 12/13 were very variable, with long indels in many sequences.

### Gap coding

Simple gap coding [50], as implemented in SeqState version 1.36, build 19.10.2007 [51], was applied to the complete alignment.

### Determination of number of sequence copies

We used a Bayesian approach to calculate the probability that we sampled all sequence copies. Let *x *be the number of sequence variants in the genome. Each PCR primer pair combination amplifies one or more sequence variants. Assuming that there is no PCR bias, the probability of sampling sequence copies will be analogous to the probability of drawing balls from a big bowl containing balls with an unknown number of different colours *(x)*.

By using a discrete uniform distribution on (1, . . ., *M*) as prior for *x*, approximating the hypergeometric distribution with a multinomial distribution with parameters (1/*x*, . . ., 1/*x*) the posterior distribution of *x *can be calculated as $\frac{{\left(1/x\right)}^{n}}{\sum_{x^{\prime }\leq x_{obs}}{\left(1/x^{\prime }\right)}^{n}}$, where *x*_*obs *_is the observed number of colours. The number of ball draws (*n*) needed for the posterior probability of *x *= *x*_*obs *_to be larger than 0.95 is given in Table 5. For the mathematical arguments, see Additional file 1. Thus, by representing PCR primer pair combinations with balls and using colours to represent paralogues, we can obtain an estimate of whether it is improbable that additional PCR primer pairs will detect additional paralogues in the genome. Cases when one primer pair results in two or more sequences will be interpreted as a draw that accidentally results in more than one ball. Note that the assumptions are that the sequences of the primer pairs are unbiased with respect to the population of sequence variants in the genome (no PCR bias). We define this population by the preliminary alignment, taken to represent the phylogenetic diversity in *Silene*. Thus, we regard sequences outside of this population as ancient outparalogues, i.e. they are not "balls". We regard this procedure as sufficient to justify the assumption that the ability of the primer pairs to amplify recent outparalogues will not, on average, be biased. Even if this assumption is overly simplistic and almost certainly violated, we think that the PCR approach employed here, with the probabilities given in Table 5, provides a useful framework for determination of the number of paralogues in a genome, when the entire genomic sequence is not known.

**Table 5:** Sample sizes needed for 95% probability to find all sequence copies

*x*_*obs*_	1	2	3	4	5	6	7	8	9
*n*	5	8	11	14	17	20	23	26	28

Sample sizes *(n) *needed for P (*x *= *x*_*obs*_) > 0.95 calculated from a discrete uniform prior of *x *on {1, 2, . . ., 10} where *x*_*obs *_correspond to the observed number of sequence variants and *n *to the number of sampled PCR fragments.

### Recombination detection

To screen for putative recombination breakpoints, GARD (Genetic Algorithm Recombination Detection) [52] was used online [53]. Due to computational limitations, a reduced alignment was analysed. In this reduced data set, only nine sequences were analysed and parts of the alignment with much missing data (first 870 bp and last 200 bp) were excluded. The XY1 introns between exons 8/9 and 12/13 were very variable in length between taxa and large parts (≈700 bp each) of these introns were also excluded (Figure 2). We used the GARD detection method using HKY85 nucleotide substitution bias model (as suggested by the model selection tool on the GARD web page [53]), with Beta-Gamma rate variation and 5 rate classes. We also tried 3–4 rate classes, and the General Discrete Distribution, with similar results not affecting the conclusions.

### Bayesian analysis

BEAST v1.4.7 [54] was used for Bayesian phylogenetic inference and dating of divergence times. Input files for BEAST were created with BEAUti v1.4.7 [54], using a relaxed clock model [55], with a Yule prior and the nucleotide substitution models proposed by MrModeltest version 2.2 [56], using the Akaike information criterion. A prior on the age of the root of the tree was set to 12.57 million years, with a normally distributed standard deviation of 2.018 [23]. Two MCMC chains were run for 10 million generations with trees and parameter values saved every 1000th generation. One of the chains had no constraints on the monophyly of the included groups, in the other chain three groups of interest were forced to be monophyletic: subgenus *Silene*, subgenus *Behenantha *(including *L. flos-jovis*) and the dioecious species of section *Elisanthe *(nested within subgenus *Behenantha*). There were no substantial differences between the two MCMC chains, except for the age of subgenus *Behenantha*. For this group, the priors had a strong impact on the results, especially when the monophyly constraints were in effect. The resulting log files were checked in Tracer v1.4 [57], and the tree files were summarized using TreeAnnotator v1.4.7 [54] into one Maximum credibility tree with median node heights (discarding the first 10% of the trees as "burn-in"). Trees were visualized using FigTree 1.1.2 [58].

### Parsimony analysis

Maximum parsimony analyses and maximum parsimony bootstrap support measures were performed with PAUP* v.4.0b10 for Unix [59] on the complete alignment with gap coding, as well as the reduced data matrix, with the data sets partitioned into the non-recombined 5' and 3' parts from the GARD recombination detection procedure. Maximum parsimony analyses were carried out using heuristic search with TBR branch swapping, multrees option in effect, and 10 random addition sequences. For bootstrap support, 1000 replicates were performed, with the multrees option off.

### Sequence divergence

Distances between the sequences were calculated using MEGA4 [60]. In addition to the pairwise distances between all sequences (data not shown), mean distances between sequence copies within an individual and mean distances between sequences from different individuals/taxa were also calculated. Divergence estimates are Tamura-Nei distances with Γ = 0.6587 and ± standard error (SE), based on 500 bootstrap replicates.

## Abbreviations

bp/kbp: base pairs/1000 base pairs; HPD: highest posterior density; MCMC: Markov Chain Monte Carlo; MPB: maximum parsimony bootstrap; PP: posterior probabilities

## Authors' contributions

AR carried out the molecular genetic studies, sequence alignment, phylogenetic analyses and drafted the manuscript. DF supplied preliminary data and helped to draft the manuscript. BS performed the statistical analysis and drafted the statistical parts of the manuscript. NH carried out parts of the molecular genetic studies (primer design, cloning) in cooperation with AR. BO conceived of the study, participated in its design and coordination and helped to draft the manuscript. All authors read and approved the final manuscript.

## Supplementary Material

Additional file 1 **Mathematical arguments for probability calculations.** The mathematical arguments used to calculate the posterior probabilities of sampling all sequence variants. Click here for file
