# Supplementary material for: Conflicting phylogenetic signals in the SlX1/Y1 gene in Silene
Source: BMC Evol Biol. 2008 Oct 30;8:299. doi: 10.1186/1471-2148-8-299 (PMC2636791; doi:10.1186/1471-2148-8-299)
Supplement: Additional file 1 — Mathematical arguments for probability calculations. The mathematical arguments used to calculate the posterior probabilities of sampling all sequence variants. [file 1471-2148-8-299-S1.pdf]

Appendix. Mathematical arguments for probability calculations

Let x be the number of sequence variants in the genome. Each PCR primer pair combination amplifies one or more sequence variants. Assuming that there is no PCR bias, the probability of sampling sequence variants will be analogous to the probability of drawing balls from a big bowl containing balls with an unknown number of different colours (x).

Suppose there are N balls in a bowl. The balls are in x different colours, each in proportion $1/x$. In a sample of n balls $\boldsymbol{k} = (k_1, \ldots, k_x)$ denotes the number of balls of colour $1, \ldots, x$. Given x, $\boldsymbol{k}$ follows a generalized hypergeometric distribution.

If N is large, the generalized hypergeometric distribution can be approximated with the multinomial distribution, that is

$$\begin{aligned} f(k_1, \ldots, k_x|x) &\simeq \begin{cases} \binom{n}{k_1 k_2 \ldots k_x} p_1^{k_1} p_2^{k_2} \cdots p_x^{k_x} & 0 \leq k_i \leq n, \sum_{i=1}^{x} k_i = n \\ 0 & \text{otherwise,} \end{cases} \\ &= \begin{cases} \binom{n}{k_1 k_2 \ldots k_x} (1/x)^n & 0 \leq k_i \leq n, \sum_{i=1}^{x} k_i = n \\ 0 & \text{otherwise,} \end{cases} \end{aligned}$$

since $p_i = 1/x$ for $i = 1, \ldots, x$.

The number of colours, x, is unknown. The Bayesian approach, with a prior $\pi(x)$ and x_{obs} the number of colours in the sample, gives the posterior distribution for x as

$$f(x|k_1, \ldots, k_x) = \frac{f(k_1, \ldots, k_x|x)\pi(x)}{\sum_{x' \geq x_{obs}} f(k_1, \ldots, k_{x'}|x')\pi(x')}, \tag{1}$$

where the sum in the denominator is over the values of x' larger than x_{obs}.

Uniform prior of x

Assume a discrete uniform prior of x on $\{1, 2, \ldots, M\}$, where M is an integer. The prior $\pi(x) = 1/M$ for all possible values of x. The formula (1) will then be

$$\begin{aligned} f(x|k_1, \ldots, k_x) &= \frac{f(k_1, \ldots, k_x|x)\frac{1}{M}}{\sum_{x' \geq x_{obs}} f(k_1, \ldots, k_{x'}|x')\frac{1}{M}} \\ &= \frac{\binom{n}{k_1 k_2 \ldots k_x}(1/x)^n}{\sum_{x' \geq x_{obs}} \binom{n}{k_1 k_2 \ldots k_{x'}}(1/x')^n} \\ &= \frac{(1/x)^n}{\sum_{x' \geq x_{obs}} (1/x')^n}, \end{aligned} \tag{2}$$

The estimate of x will be $\hat{x} = \text{argmax}_i\, f(x_i|k_1, \ldots, k_{x_i}) = x_{obs}$.

Sample size

How large sample is needed for the posterior probability of $x > x_{obs}$ to be small enough? Since $\hat{x} = x_{obs}$ and $P(x < x_{obs}) = 0$, this is equivalent to the posterior probability of $x = x_{obs}$ being large enough, which can be calcuated from (2).

Table 1 give the sample sizes needed for the posterior probability of $x = x_{obs}$ to be larger than 0.95, given a uniform prior of x on $(1, \ldots, 10)$.

Table 1: *Sample sizes needed for* $P(x = \hat{x}) > 0.95$ *calculated from a discrete uniform prior of* x *on* $\{1, 2, \ldots, 10\}$.

$\hat{x}$	1	2	3	4	5	6	7	8	9
n	5	8	11	14	17	20	23	26	28

In real data, x_{obs} is often small. Calculating the sample sizes needed for $P(x = \hat{x}|\hat{x}) > 0.95$ for $x_{obs} \in \{1, 2, 3, 4\}$ and $M = 5$ gives the same sample sizes as in Table 1. Letting M increase a priori does not change the sample sizes needed. The reason is that (2) will be affected only in the denominator by adding terms $(1/i)^n$ in the sum. Denote $s_m = \sum_{i=1}^{\infty}(1/i)^m$. It is known that (see e.g. Beta Mathematics Handbook [63]) $s_3 \simeq 1.2021$, $s_5 \simeq 1.0369$ and

$$s_{2n} = \frac{2^{2n-1}\pi^{2n}}{(2n)!}(-1)^{n-1}B_{2n}, \tag{3}$$

where B_i =Bernoulli numbers. ($B_8 = -1/30$, $B_{14} = 7/6$). Therefore, $P(x = \hat{x}|\hat{x} = 1)$, will, as $M \to \infty$ and $n = 5$ be

$$\begin{aligned} P(x = \hat{x}|\hat{x} = 1) &= \frac{1}{\sum_{x'=1}^{\infty}(1/x')^n} \\ &= \frac{1}{s_5} \\ &\simeq \frac{1}{1.0369} > 0.95. \end{aligned}$$

For $\hat{x} = 2$ we can calculate the posterior probability of $P(x = \hat{x}|\hat{x} = 2)$ as $M \to \infty$ and $n = 8$ as

$$
\begin{aligned}
P(x = \hat{x}|\hat{x} = 2) &= \frac{(1/2)^n}{\sum_{x'=2}^{\infty}(1/x')^n} \\
&= \frac{(1/2)^8}{s_8 - 1} \\
&= \frac{(1/2)^8}{\frac{2^7\pi^8}{(8)!}(-1)^7 B_8 - 1} \\
&= \frac{(1/2)^8}{\frac{2^7\pi^8}{(8)!}(-1)^7(-1/30) - 1} \\
&> 0.95.
\end{aligned}
$$

In the same way we can conclude that $P(x = \hat{x}|\hat{x} = 4) > 0.95$, using $n = 14$, B_{14} and $M \to \infty$. For $\hat{x} = 3$ we use $s_{11} \simeq 1.000494$, calculated with 3740 terms in the sum, the next term is less than $5 \cdot 10^{-40}$. Using the same kind of calculations as above, the sample size $n = 11$ is enough for the posterior probability of $x = \hat{x}$ to be larger than 0.95. We therefore conclude that the sample sizes needed are robust for $M \geq 5$.
